# Supplementary material for: Modeling glioblastoma heterogeneity as a dynamic network of cell states
Source: Mol Syst Biol. 2021 Sep 16;17(9):e10105. doi: 10.15252/msb.202010105 (PMC8444284; doi:10.15252/msb.202010105)
Supplement: Supplementary file 6 — Source Data for Figure 5 [file MSB-17-e10105-s004.zip › Figure5A_sourcedata/GSEA_3071/nosigdb_stateI.GseaPreranked.1622795145664/SEGERMAN_MULTITHERAPY_RESISTANCE_FIG5A_PMID_27974212.html]

Details for gene set SEGERMAN\_MULTITHERAPY\_RESISTANCE\_FIG5A\_PMID\_27974212[GSEA]

|  || Dataset | state13071 |
| Phenotype | NoPhenotypeAvailable |
| Upregulated in class | na\_pos |
| GeneSet | SEGERMAN\_MULTITHERAPY\_RESISTANCE\_FIG5A\_PMID\_27974212 |
| Enrichment Score (ES) | 0.6656172 |
| Normalized Enrichment Score (NES) | 2.6954422 |
| Nominal p-value | 0.0 |
| FDR q-value | 0.0 |
| FWER p-Value | 0.0 |
Table: GSEA Results Summary

  

Fig 1: Enrichment plot: SEGERMAN\_MULTITHERAPY\_RESISTANCE\_FIG5A\_PMID\_27974212      
 Profile of the Running ES Score & Positions of GeneSet Members on the Rank Ordered List

  

| PROBE | GENE SYMBOL | GENE\_TITLE | RANK IN GENE LIST | RANK METRIC SCORE | RUNNING ES | CORE ENRICHMENT || 1 | FN1 |  |  | 11 | 1.769 | 0.1153 | Yes |
| 2 | EDIL3 |  |  | 62 | 1.251 | 0.1847 | Yes |
| 3 | PPIC |  |  | 77 | 1.195 | 0.2607 | Yes |
| 4 | AHNAK |  |  | 163 | 0.887 | 0.2957 | Yes |
| 5 | EMILIN1 |  |  | 207 | 0.812 | 0.3377 | Yes |
| 6 | MSRB3 |  |  | 242 | 0.743 | 0.3777 | Yes |
| 7 | SCPEP1 |  |  | 259 | 0.727 | 0.4218 | Yes |
| 8 | DAB2 |  |  | 277 | 0.702 | 0.4640 | Yes |
| 9 | CLMP |  |  | 307 | 0.665 | 0.5002 | Yes |
| 10 | SHC1 |  |  | 361 | 0.624 | 0.5268 | Yes |
| 11 | EPHX1 |  |  | 429 | 0.570 | 0.5457 | Yes |
| 12 | FSTL1 |  |  | 505 | 0.504 | 0.5579 | Yes |
| 13 | IGDCC4 |  |  | 517 | 0.497 | 0.5880 | Yes |
| 14 | BLVRB |  |  | 535 | 0.488 | 0.6159 | Yes |
| 15 | SLC46A3 |  |  | 621 | 0.443 | 0.6212 | Yes |
| 16 | COPZ2 |  |  | 650 | 0.432 | 0.6421 | Yes |
| 17 | SLC2A10 |  |  | 760 | 0.394 | 0.6372 | Yes |
| 18 | OSTF1 |  |  | 808 | 0.378 | 0.6491 | Yes |
| 19 | LOXL1 |  |  | 857 | 0.363 | 0.6596 | Yes |
| 20 | SERPINB1 |  |  | 918 | 0.346 | 0.6656 | Yes |
| 21 | TBX15 |  |  | 1074 | 0.312 | 0.6420 | No |
| 22 | LAMA5 |  |  | 1087 | 0.310 | 0.6593 | No |
| 23 | TRIM16 |  |  | 1334 | 0.262 | 0.6064 | No |
| 24 | TMBIM1 |  |  | 1339 | 0.261 | 0.6227 | No |
Table: GSEA details [plain text format]

  

Fig 2: SEGERMAN\_MULTITHERAPY\_RESISTANCE\_FIG5A\_PMID\_27974212: Random ES distribution      
 Gene set null distribution of ES for **SEGERMAN\_MULTITHERAPY\_RESISTANCE\_FIG5A\_PMID\_27974212**
